# Tissue engineering of the larynx: A contemporary review

**DOI:** 10.1002/jcla.23646

**Published:** 2020-12-15

**Authors:** Jingjing Chen, Yi Shen, Zhisen Shen, Lixin Cheng, Shuihong Zhou

**Affiliations:** ^1^ Department of Otorhinolaryngology–Head and Neck Surgery Lihuili Hospital Ningbo University Ningbo 315040 China; ^2^ Department of Otorhinolaryngology– Head and Neck Surgery The First Affiliated Hospital College of Medicine Zhejiang University Hangzhou 310003 China

**Keywords:** biomolecules, cartilage regeneration, laryngeal carcinoma, scaffolds, stem cells

## Abstract

**Objective:**

Tissue engineering has been a topic of extensive research in recent years and has been applied to the regeneration and restoration of many organs including the larynx. Currently, research investigating tissue engineering of the larynx is either ongoing or in the preclinical trial stage.

**Methods:**

A literature search was performed on the Advanced search field of PubMed using the keywords: “(laryncheal tissue engineering) AND (cartilage regeneration OR scaffolds OR stem cells OR biomolecules).” After applying the selection criteria, 65 articles were included in the study.

**Results:**

The present review focuses on the rapidly expanding field of tissue‐engineered larynx, which aims to provide stem cell–based scaffolds combined with biological active factors such as growth factors for larynx reconstruction and regeneration. The trend in recent studies is to use new techniques for scaffold construction, such as 3D printing, are developed. All of these strategies have been instrumental in guiding optimization of the tissue‐engineered larynx, leading to a level of clinical induction beyond the in vivo animal experimental phase.

**Conclusions:**

This review summarizes the current progress and outlines the necessary basic components of regenerative laryngeal medicine in preclinical fields. Finally, it considers the design of scaffolds, support of growth factors, and cell therapies toward potential clinical application.

## 1 INTRODUCTION

Laryngeal carcinoma is the second most common head and neck cancer, and occurs more commonly in men than in women.^1^ With an estimated incidence rate of 5.8/100 000 in males, it can seriously threaten health and quality of life.^2^ Approximately 60% of patients initially present with an advanced primary tumor (stage III or IV) and, once diagnosed, usually portend a poor outcome and lower treatment efficacy. Despite continuing efforts to improve/optimize outcomes in individuals with laryngeal carcinoma and preserve laryngeal function through radiation‐based strategies, there are limited therapeutic options.^3^, ^4^ For advanced primary tumor(s) or previously treated dysfunctional larynx, total or partial laryngectomy plays a critical role and remains the primary method of treatment.^5^ These surgical methods influence the capacity of phonation and airway protection during swallowing.^6^ The need for laryngectomy persists among individuals with a dysfunctional larynx and poor quality of life.

Dysfunctional larynx can lead to problems with speech, breathing, swallowing, taste, and smell. However, surgery can result in significant—if not traumatic—changes to cosmetic appearance, which can be devastating to some patients. Loss of a functioning larynx also heavily impacts social functioning and the ability to work. To address these problems, allograft transplantation of laryngeal tissue was attempted in 1969.^7^ Although the attempt was unsuccessful, it prompted more clinical research. However, to our knowledge, only two successful laryngeal allotransplantations have been reported to date.^8^, ^9^ The disadvantages of laryngeal transplantation include risks for reperfusion injury and infection. Several problems, such as ethical concerns and lifelong immunosuppression, are associated with such a procedure.^10^ Moreover, functional integration with the nerve‐muscle unit after allotransplantation is not currently possible.

As a rapidly expanding field, tissue engineering is reaching maturity and yielding promising outcomes.^11^ Previous research investigating tissue engineering of the larynx aims to improve functionality post‐laryngectomy and reconstruct damage without the need for subsequent immunosuppression. It is more likely that restoration of laryngeal defect(s) in the future will be based on tissue engineering methods rather than allotransplantation. However, to tissue engineer a functional larynx successfully, an understanding of its normal anatomy and physiology is required.

Anatomically, the larynx is a hollow, three‐dimensional structure consisting of thyroid, ring‐shaped, arytenoid, and epiglottic cartilages. The cartilage in the laryngeal cavity is connected by muscles. Muscle relaxation and contraction can control the tension of the vocal cords, as well as opening and closing of the glottis.^12^ Due to its special location, function, and natural morphology, tissue engineering of the larynx presents significant challenges but also particular advantages in reconstruction.^13^ The study of cartilage tissue engineering has an important role to play in rebuilding and shaping the head and neck, as well as reconstituting, specifically in larynx cartilage reconstruction.^14^, ^15^ For the past few decades, many research teams have used an excessively empirical approach to cartilage repair; however, they now tend to focus on a more biological approach using novel tissue engineering–based strategies.^16^

In the past two decades, three key elements have formed the building blocks of the tissue engineering–based approach: a matrix scaffold, cells sources, and growth factors (or genetic regulators).^17^, ^18^ The optimal tissue–engineered laryngeal cartilage with good biocompatibility and biodegradability requires a three‐dimensional scaffold and a large quantity of cells and signaling molecules.^19^ The following sections describe the key constituents of a tissue engineering–based approach to laryngeal cartilage repair.

## 2 CURRENT RESEARCH ACTIVITIES RELATING TO TISSUE‐ENGINEERED LARYNX

### 2.1 Scaffold

The first case to use a scaffold as a cell carrier in cartilage repair dates back to the 1960s.^20^ Since then, synthetic polypropylene mesh scaffolds have been used to achieve partial laryngeal replacement in pigs.^21^, ^22^, ^23^ To mimic the native larynx, biomaterials amenable to shaping, with specific mechanical strength, flexibility, biocompatibility, and biodegradability, are needed, not only in vitro but also in vivo, for their capacity to facilitate laryngeal cartilage reconstruction. These biomaterials can be broadly divided into two categories—natural and synthetic—which are discussed below.

Natural materials with suitable bioengineering characteristics in regulating cell response(s) include carbohydrate‐based polymers (eg, polylactic acid, polyglycolic acid, hyaluronan, agarose, alginate, and chitosan) and protein‐dependent polymers (eg, fibrin, gelatin, and collagen), which are generally used in cartilage repair.^24^ Some evidence supports agarose as a potential scaffold candidate because it has been used as a matrix in cartilage tissue engineering owing to its high water absorbance capacity, similar to the extracellular matrix (ECM).^25^ Similar to agarose, alginate enables maintenance of the chondrocytic phenotype and has been extensively used in tissue engineering as a cartilage substitute owing to its biocompatibility and non‐immunogenicity.^26^ Many studies have demonstrated the chondrogenic potential of alginate scaffolds.^27^, ^28^ Human fibrin gels, which are Food and Drug Administration‐approved materials, exert a pro‐inflammatory effect and induce their own degradation by components of the ECM into nontoxic endpoint components. The use of fibrin glue and chondrocytes improve the repair of cartilage in vivo.^29^ As a natural protein, collagen serves as a scaffold substitute, with good cell adhesion properties, and supports chondrocyte proliferation in vivo.^30^ Miao et al^31^ reported that collagen scaffolds can improve the process of spontaneous repair of osteochondral defects better than other hydrogels.

In addition to natural materials, synthetic materials have several potential advantages including biocompatibility, low toxicity, and excellent mechanical properties.^32^ Different types of synthetic materials are used in engineering fields, namely Dacron (polyethylene terephthalate), Teflon (polytetrafluoroethylene), carbon fiber, polyester urethane, polybutyric acid, polyethyl methacrylate, and hydroxyapatite.^17^ Polyethylene glycol is chemically synthesized to act as a supporting agent in cartilage tissue engineering with good biocompatibility and hydrophilicity.^33^ Polylactide acid (PLA) and poly(lactic‐co‐glycolic acid) have been described as potential scaffold materials that promote cell proliferation and differentiation in cartilage tissue engineering.^34^ The main disadvantage of PLA is its cytotoxicity and potential to elicit immunological reactions.^35^ Although some of the listed materials are already in clinical use, most are still being tested in preclinical trials.^36^

Using three‐dimensional printing technology, the electrospinning technique and nanotechnology aim to create an absorbable and biomimetic scaffold and stimulate the extracellular microenvironment of the native cartilage.^37^, ^38^ At the nanoscale level, the interaction between scaffolds and cells becomes more active owing to the unique features of nanomaterials compared with larger‐scale materials. In turn, this enhances cell behavior to a significant extent, resulting in changes in cell shape and motility, along with the expression of different genes.^39^

### 2.2 Cell sources

Ideally, cell‐based tissue‐engineered laryngeal cartilage would have cells evenly distributed throughout the scaffold, which would fuse with the adjacent tissue (ie, laryngeal muscle) without inducing an inflammatory response. Cell‐based therapies have been shown to repair partial laryngeal defects in vivo.^40^ Although autologous chondrocyte implantation is used to repair laryngeal cartilage defect(s) with good results, the main drawbacks are biological and surgical limitations.^41^

The search for ideal cell sources has attracted attention to the field of cartilage regeneration as a new powerful tool in scaffold augmentation.^42^ Precursor cells of different tissue origins exist in adult mammals and can be used for transplantation purposes. Mesenchymal stem cells (MSCs) are primitive precursor cells that give rise to multiple cell types including osteoblasts and chondrocytes owing to their capacity for self‐renewal and accessibility.^43^ Other cell types, such as perinatal cells, embryonic stem cells, and chondroblasts, also have the potential to differentiate into cartilage.^44^ Some notable cartilage engineering in the field of otolaryngology includes the research by Zhang et al, who created three‐dimensional tissue‐engineered laryngeal cartilage from adipose‐derived MSCs (ADMSCs) in vivo.^40^ However, the most significant disadvantage is that the chondrogenic potential of bone marrow–derived mesenchymal stem cells (BMSCs) declines with age.^45^

There have been only a few studies investigating synovium‐, peripheral blood‐, and umbilical cord blood–derived MSCs, ^46^ and it remains to be confirmed whether induced pluripotent stem cells can differentiate and mature into cartilage tissue.^47^ Many existing problems with stem cells, such as age, maturation state, newly formed cells, and tissue matches with the donor, need to be resolved. The most prominent challenge in the use of stem cells for differentiation into chondrocytes is avoiding hypertrophy, which demands biological, chemical, and physical regulation.^48^ Ongoing studies continue to search for the ideal source of MSCs suitable for the clinical repair of the laryngeal cartilage.

### 2.3 Biomolecules

In addition to creating tissue‐engineered laryngeal cartilage, successful regeneration of the laryngeal cartilage tissue not only relies on the scaffold and cells, but is also significantly influenced by the microenvironment in which cells grow.^49^ Biomolecules include growth, differentiation, angiogenic, and gene‐modulated factors, which play important roles in the microenvironment. Similar to the ECM, biomolecules have a powerful influence on the migration, differentiation, and proliferation of cells.^50^, ^51^ To optimize differentiation, it is essential to use well‐characterized growth factors.

The main growth factors include transforming growth factor‐beta (TGF‐β), insulin‐like growth factor‐1 (IGF‐1), bone morphogenetic proteins (BMPs), platelet‐derived growth factor (PDGF), vascular endothelial growth factor (VEGF), epidermal growth factor (EGF), and fibroblast growth factor (FGF)‐2. TGF‐β is a multifunctional factor in the mitogenic process that controls proliferation and differentiation of many cell types and may enhance the activity of PDGF, b‐FGF, and EGF.^52^ IGF‐1 has demonstrated potential in cartilage grafting proliferation and peripheral nerve regeneration, which also stimulate the differentiation of MSCs in chondrogenesis.^53^, ^54^ Released from activated platelets, PDGF is involved in inflammatory responses, reconstructive processes, and hemostasis.^55^ It induces collagen biosynthesis and angiogenesis as a mitogenic and chemotactic factor.^56^, ^57^ BMPs act as a key factor in osteogenesis and osteoinductively influence regeneration of the cartilage directly and indirectly, and stimulate the differentiation of MSCs into various cell types.^58^

In most cartilage‐engineering strategies, many elements influence the efficacy of biomolecules, including cell stage and treatment dose and duration.^59^ This has been evaluated mainly in vitro and to only a limited degree in vivo (only TGF‐β has been shown to be effective).^60^ Nevertheless, future research will focus on testing small signal molecules that exert a generalized anabolic effect on chondrocytes.

## 3 FUTURE WORK

Research in laryngeal tissue engineering was hardly existent until the turn of the 21st century. The number of publications describing tissue engineering larynx has been rapid growth since that time (Table 1). Although still in its infancy, research activity investigating the application of laryngeal tissue engineering in reconstructive medicine suggests rapid advances and developments in the future. The aim of laryngeal tissue engineering is to develop methodologies by which laryngeal defects can be repaired and demonstrate its potential to transform clinical care. Although translational progress remains in the early stages, it is appropriate to assess strategic directions in laryngeal tissue engineering.

**Table 1:** Summary of the studies of tissue‐engineered larynx

Author	country	Year	Model	Scaffold	Cell sources	Assessment	Reference
Herrmann P et al	Britain	2017	Pigs	Decellularized larynx	Human BM‐MSC	In vivo	[23]
Sun A et al	China	2015	Rabbits	Porous PHBHH	Costal and articular chondrocytes	In vitro	[22]
Gilpin DA et al	America	2010	Rabbits	Scaffold free	Autologous auricular chondrocytes	In vivo	[41]
Ansari T et al	Britain	2017	Pigs	Decellularized hemilarynges	Human BM‐MSC	In vivo	[63]
Kamil SH et al	America	2004	Pigs	Polymer (Pluronic F‐127)	Autologous auricular chondrocytes	In vivo	[24]
Zhang H et al	India	2017	Rats	Collagen oligomer	Autologous ASCs	In vitro and in vivo	[40]
Jotz GP et al	Brazil	2017	Pigs	Poly‐DL‐lactide	Human MSCs	In vivo	[61]
Omori K et al	Japan	2008	Human	Collagen sponge	Cell free	In vivo	[21]

**Figure 1:**
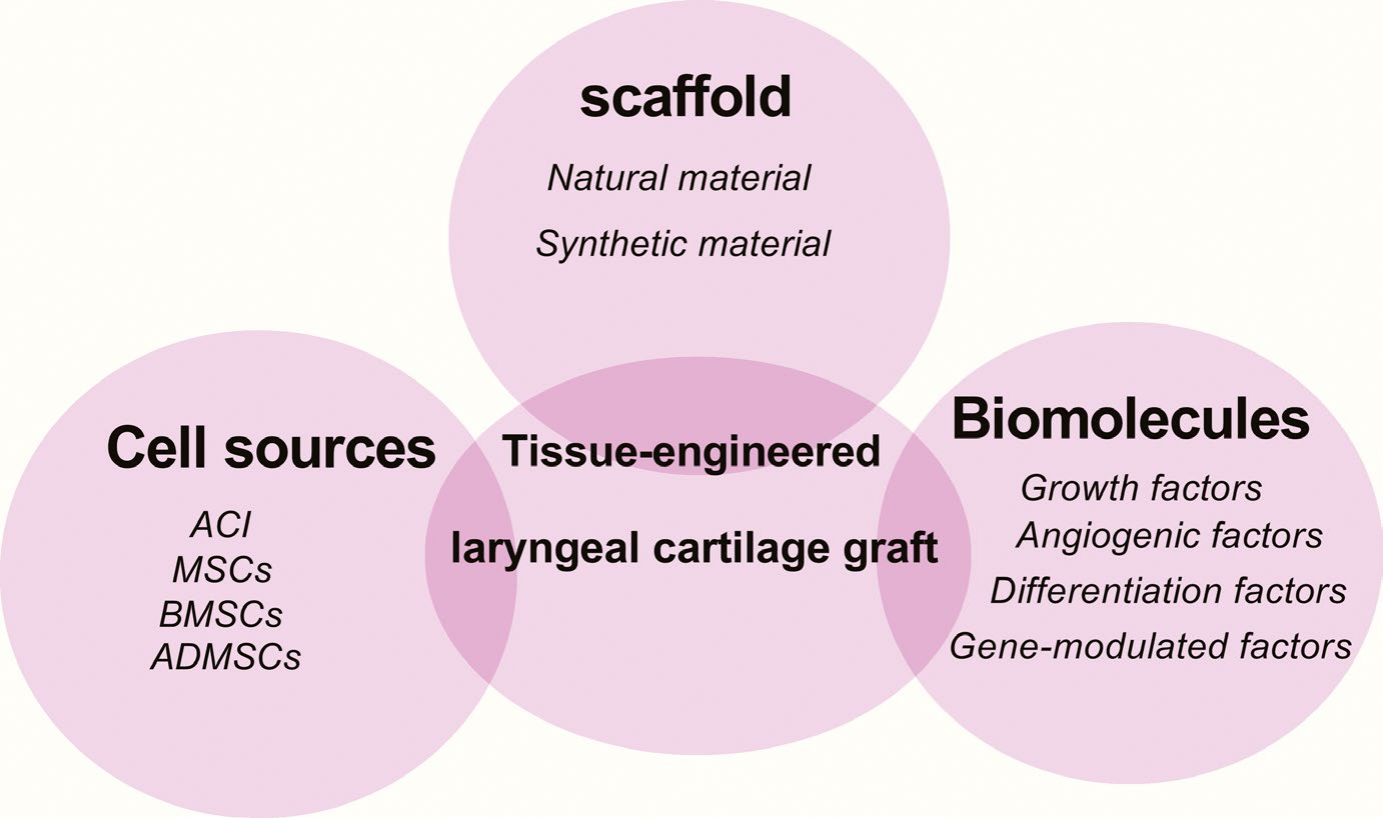
Tissue engineering–based approach to tissue‐engineered repair

An ideal tissue‐engineered constructs should mimic the internal environment and maintain the mechanical properties of the larynx. Due to special characteristics of the larynx, it is difficult to develop a tissue‐engineered larynx without suitable scaffolds, cells, and growth factors (Figure 1). Most of the experience so far gained with tissue‐engineered constructs has focused on biocompatible and nontoxic scaffold without cells, although cells play important roles. The “ideal” cell type must be sufficient and functional for the purpose of harvesting without infection, immune response, and possible tumor formation. This may be more technically challenging in practice. Irrespective of the type of cell that is employed, cell choice, isolation, and seeding must be concerned before the goal of larynx cartilage regeneration can be achieved. Whether to seeding remains controversial; however, the majority of research has demonstrated partiality toward seeded scaffolds. Jotz et al reported that MSC scaffolds demonstrated a significant advantage in forming laryngeal neo‐cartilage in a porcine model.^61^ The seeded cells may act as a “feeder layer” by activating local progenitor cells and accelerating the process of tissue integration.^62^ Although not formally assessed, Herrmann et al^23^ demonstrated that each animal had normal respiratory, sounding, and swallowing functions post‐surgery of the larynx, without adverse clinical effects due to an implanted and seeded decellularized scaffold.

Different from other kinds of cartilage regeneration, the construction of tissue‐engineered laryngeal cartilage has intricate cartilaginous complex in keeping the airway patent demanding for biochemical and material properties. In laryngeal biology, what is needed now is a new concept of cartilage repair, cutting‐edge techniques, and systematic strategies for evaluation. The implanted scaffold provides a skeletal frame for larynx regeneration. To create a suitable scaffold with a hollow structure, such as the larynx, choosing “smart” materials with excellent biocompatibility and biodegradability with minimal side effects to mimic the structure of native laryngeal cartilage is the key to improving laryngeal cartilage reconstruction.

## 4 PERSPECTIVES

It is not currently possible to replace the entire larynx with fully vascularized, nerve innervated, tissue‐engineered products. Laryngeal regenerative medicine is currently focused on replacing the hemi‐larynx as opposed to the entire larynx, while maintaining fundamental functions, such as respiratory function, in preclinical studies. Ansari et al implanted a porcine hemi‐larynx into a porcine animal model to complete epithelialization of the mucosal surface without previous attempts at vascularization of the scaffold.^63^ Despite advances and rapid development in cartilage tissue engineering, functional repair using tissue‐engineered laryngeal cartilage has not yet been reported in the clinic. The main problems include vascularization, mucosalization, and support in cartilage reconstruction for laryngeal cartilage. According to the common nearest transfer, using a tissue flap to provide a blood supply and subsequently preparing for the tissue‐engineered cartilage may overcome the problems with vascularization.^64^ To complete mucosal coverage and heal the damaged cartilage, laryngeal replacement needs to use cells and/or growth factors to inhibit tissue scarring accompanied by inflammatory responses, including neutrophil infiltration, together with calcification.^65^

The present review highlights the promising future of tissue‐engineered laryngeal cartilage; however, there is still a need for comprehensive development of cutting‐edge techniques, especially in three‐dimensional printing (3D), 4D printing (3D printing of programmable inks), or 5D printing as a five‐axis system for printing complex structures in multiple dimensions of tissue scaffolds for futuristic tissue engineering and regenerative medicine. A major advantage of tissue‐engineered laryngeal cartilage would be the facilitated differentiation of host and donor cells. Disadvantages of grafting tissue–engineered laryngeal cartilage include the high costs and the time interval for the growth of an adequate and useable piece of cartilage. Further experimental studies and development of surgical procedures are needed to validate the use of tissue‐engineered laryngeal scaffolds.

## 5 CONCLUSION

Despite considerable technical obstacles, there have been rapid advances and developments in laryngeal tissue engineering. The technology of tissue‐engineered larynx combined with experimental developments will improve survival and surgical outcomes in the field of laryngeal diseases. Of the proposed approaches, research focused on laryngeal studies in humans is expected in the future.
